# Inpatient end-of-life care delivery: discordance and concordance analysis of Canadian palliative care professionals’ and South Asian family caregivers’ perspectives

**DOI:** 10.1177/26323524221145953

**Published:** 2023-01-11

**Authors:** Swarna Weerasinghe

**Affiliations:** Department of Community Health and Epidemiology, Faculty of Medicine, Dalhousie University, Centre for Clinical Research, 5790 University Avenue, Halifax, NS B3H 4H7, Canada

**Keywords:** discordance and concordance analysis, end-of-life care, inpatient palliative care, palliative healthcare professional perspectives, South Asian family caregiver perspectives

## Abstract

**Background:**

End-of-life care involves a multitude of functions delivered by a team of healthcare professionals. Family caregivers get involved in every aspect of the palliative care journey. Meeting the needs of ethnically diverse patients can be a daunting task for Western-trained healthcare professionals. Family and professional caregivers need to have a mutual understanding of perspectives and expectations to integrate family caregivers into end-of-life care. The South Asian population in Canada is fast growing, and very little is known about their understanding and expectations of end-of-life care.

**Methods:**

The purpose is to provide research-based knowledge on discordances and concordances of encounters and perceptions of end-of-life care delivery between South Asian family caregivers and palliative care health professionals. Individual interviews were conducted among seven palliative care professionals, in a tertiary care center, and seven South Asian family caregivers who have provided care, in the same inpatient center, for the same period. The constant comparison, a component of the grounded theory approach, was employed to compare the two types of caregivers’ perspectives that emerged in the qualitative data.

**Findings:**

The family caregivers were divided in their perception based on death denial and acceptance. The findings weaved the discordances and concordances of meaning assigned to palliative care to the three themes that emerged: the role of the family caregiver, communication needs and challenges, and barriers to the family caregiver participation in decision-making. The discordance between professionals and family caregivers arose in the death-denial group and concorded with the death-accepted group. The findings revealed a consequence of the survival optimistic bias, as creating dissatisfaction toward the end-of-life care delivery system when the palliative care professionals prognosticate imminent end-of-life.

**Conclusion:**

The family caregivers’ interactions and encounters were shaped by their acceptance or denial of the death of their family member in care. Gaining conceptual clarity on the meaning of palliative care and providing education on the process of end-of-life care delivery are crucial to integrating ethnically diverse family caregivers into the decision-making process.

## Background

The components of palliative care (PC), integral to end-of-life care (EoLC), encompass acknowledging the inevitability of death, meeting patient and family expectations, understanding patient and family preferences, facilitating access to spiritual and emotional support, and providing the best possible treatment and care (though not lifesaving) to ensure comfort and a sense of control.^1–4^ EoLC involves a multitude of functions, physical, spiritual, and psychosocial assessment, care, and treatment, delivered by health professionals and ancillary staff.^5^ The team of healthcare professionals, who carried out these functions, include PC physicians, nurses, and social and spiritual care workers. Irrespective of ethnic background, family caregivers (FCs) get involved in every aspect of the PC journey and ethnic minority patients rely on more informal support from their families than from PC health professionals.^6^ Meeting wider spectra of the needs of ethnically diverse non-Western patients, within each of those EoLC functions, is a daunting task for healthcare professionals, who operate in a system grounded in Western biomedical practices. To facilitate the needs and the process, the social and spiritual care workers act as a bridge between the FCs and healthcare professionals,^7^ and they need to be equipped with the necessary knowledge of cultural variations to emphasize patient-centered care.^8^ PC is grounded in patient-centered care, which is responsive to individual patient values and needs and incorporates these in clinical decision-making.^5^ Even further, patient-centered care is emphasized in the PC literature to highlight the need to facilitate a personalized approach, supporting patients to effectively navigate symptom management and prognostic uncertainties.^9^ The advantages of patient-centered EoLC include optimization of quality of life by prognosticating, preventing, and treating pain and suffering.^10^

The far-reaching goal of this study is to facilitate South Asian (SA) FCs becoming an integral part of an inpatient PC team delivering care to their loved ones. This can be achieved by both palliative care health professionals (PCHPs) and SA FCs knowing similarities and differences in their expectations, understandings, and perspectives of EoLC. This article aims to provide research-based knowledge on discordances and concordances of experiences, understandings and perceptions of PC, and related encounters of EoLC delivery between SA FCs and PCHPs. Although the SA population in Canada is growing and is the second-largest aging population in Canada,^11^ there is a dearth of research-based knowledge on their understanding and expectations of EoLC that are similar and different from PCHPs.

### PC and EoLC: definitions and Canadian frameworks

EoLC, the last component of PC, is offered to patients with a condition of high mortality risk.^12,13^ The World Health Organization (WHO) refers to PC as relieving pain and suffering, while improving quality of life.^14^ In this article, PC is defined as the care that focuses on providing comfort care and supportive physical, psychological, social, and spiritual care to individuals suffering from life-limiting conditions, during the time of EoL, and extended comfort care provided to the family through the bereavement process. Within this definition, social and spiritual care workers’ support provided to the bereaved family members is also considered a part of PC.^15^ The composite role of the social worker in PC includes providing psychosocial assessment and delivery of interventions and coordination of care delivery while acting as a bridge between the patient and the rest of the PC team.^16^ The spiritual care workers organize and respond to patient and family spiritual beliefs and rituals at EoL. Although there is no agreement in the literature on what stage of dying constitutes EoLC, a Cochrane review that focused on clinical pathways suggested the care delivered in the last few days of the PC journey be considered EoLC.^17^

### EoLC delivery to ethnically diverse populations

Ethnic immigrant populations, characterized by their place of birth, hold diverse and competing cultural practices and value systems that govern their expectations of EoLC services.^18^ Scholars have recognized the challenges faced by PCHPs in balancing the cultural values and beliefs, of non-Western patients and families, with those of the EoL healthcare delivery practices in Western countries.^19^ A US study among Black and White community-dwelling adults identified cultural values, beliefs, and communication patterns that could promote cultural competency among PCHPs.^6^ Besides identification, research-based evidence has revealed the effectiveness of the existing practices, tools, and guidelines in the United States, in delivering culturally competent patient-centered EoLC to ethnic minorities by social workers.^13^ Even further, the National Association of Social Work in the United States emphasized the need for acknowledging and acting on ethnocultural traditions and values systems related to end-of-life, as a standard of practice.^20^ Moreover, the literature emphasized the crucial role played by inpatient PC team members and considers them as the backbone of the PC.^21^

Previously, a study conducted among SA FCs in the study area of Halifax, Nova Scotia, Canada, identified two areas of constraints to optimal access to inpatient EoLC: language and communication barriers and lack of acculturation to and familiarity with the Western biomedical PC system.^18^ This study aims to further the existing knowledge by exploring the dual perspectives, uncovering PCHP encounters of EoLC delivery to ethnically diverse SAs, in parallel to their FCs’ experience, of the same encounters, in the same inpatient PC care setting.

Two literature reviews employed concordance and/or discordance on perceptions of EoLC between professional caregivers and FCs. One scoping review compared research-based knowledge, of perceptions of FCs, PCHPs, and patients, coming from 21 different study settings and geographies.^5^ The second review synthesized discordance and/or concordance in EoLC decision-making between FCs and patients, and their findings are not directly comparable with our study findings.^22^ Our analysis took a slightly different stance, wherein the contents related to concordance and discordance between SA FCs and PCHPs who provided care to the same patient population were analyzed in the same inpatient care setting in parallel and subsequently compared within the same emerging theme. The findings provided specific directions to find common grounds to integrate FCs more effectively into the EoLC planning.

## Methods

### Study design

A qualitative research design based on grounded theory^23^ was employed in the data collection and analysis. Seven individual interviews were conducted among SA FCs, who met the inclusion criteria of having provided EoLC for a family member, while hospitalized in the PC unit, within 10 years of the date of data collection. The FC study participants’ inclusion criteria included having participated in EoLC delivery to a deceased family member who received PC in the study tertiary care center, PC unit, in Nova Scotia, Canada. The study participants, PCHPs, were working in the same PC facility. Snowball and purposive sampling strategies were used to reach out to SA FCs. We approached prospective participants through online SA community association contacts, and PCHPs were reached through hospital contacts. A semi-structured interview guide was used for data collection (Table 1). All interviews were conducted in English. The PC unit in the study area hospital is small, and we had difficulties in recruiting more than seven PCHP study participants, who met the inclusion criteria of having given care to SA patients within the last 10 years. The duration of 10 years was chosen to include more study participants because our initial duration of giving care within 5 years resulted in three PCHPs. In this article, the constant comparison method of analyzing interview data from two different groups, PCHPs and FCs, was applied.

**Table 1.** Interview guide questions.

Topic	Family caregiver questions	Professional caregiver questions
Meaning of palliative care	• What does palliative care mean to you?	• What does palliative care mean to you?
Role of culture/ethnicity in end-of-life care	• What role did cultural beliefs play in your loved one’s end-of-life care? • Probes: What role did spirituality/religion play in your loved one’s end-of-life care? • What would have made a difference in his/her death, if you were he/she was in the county of birth? • Is there anything specific about death and dying rituals (from your cultural and religious view) that you would have wanted your caregiver to know or understand?	• What do you find most challenging about your work? • Describe your experience. (If more than one, then describe as many as possible) Probes: How long was the individual in your care? *Who else was involved in the care of this person (e.g. family, friends, etc.) *Did you experience communication barriers in relaying information to the patient and family? How were the language barriers accommodated? *Did the person have any unique needs, for example, dietary needs, religious/cultural/spiritual needs, alternative healing practices, etc.? *How were these needs accommodated? *What did you find most challenging about caring for this patient(s) *What information do you currently have to prepare you for caring for a South Asian patient and their family? *What would you like to know about South Asian cultures?
Caregiver expectations/expectations	• What would you have wanted your professional caregiver to know about your culture or religion? For example, diet, physical comfort, clothing, religious practices, pain relief, etc. • Describe cultural conflicts and language and cultural barriers that you experienced. What worked well?	• Did South Asian end-of-life patients have unique religious/cultural/spiritual and language needs? • What suggestions do you have to make the healthcare system more culturally sensitive?

### Setting

This study focused on one of the EoLC delivery models practiced in Canada, the inpatient PC consultation services, and treatment delivery in hospital and acute PC units. Although the study setting includes EoLC delivery in an acuity PC unit, the study focused on acuity and nonacuity inpatient EoLC delivery, a provision of care that requires greater interaction of PCHPs with FCs than those who receive outpatient, community-based, and hospice EoLC. The study setting is the PC unit in the study tertiary care center, in Nova Scotia, Canada.

This research project received ethics approval from the Capital District Health Authority, Research Ethics Board, and Dalhousie University Health Sciences Ethics Review Board.

### Data collection and analysis

Written informed consent was obtained before each interview. The interview guides and the consent forms were approved by the Capital District Health Authority, Research Ethics Board, and Dalhousie University Health Sciences Ethics Review Board.

Seven individual interviews were conducted among PCHPs, including PC nurses and doctors, spiritual care providers, and social workers. The interview guide was created based on researchers’ experience in the interpretation of diverse meanings assigned to health by immigrant populations (question 1, Table 1) and the role-play of culture and religion in health.^24^ Interview guide for PCHPs was reviewed by the head of the PC unit. At the end of each interview, we administered a survey to collect basic demographic information about the study participants. The interview data were audio-recorded and transcribed verbatim and analyzed by two qualitative methodologists. FC data were coded immediately after collection and transcription, and the point of saturation was determined when no new codes emerged, which happened at the sixth interview and one additional interview was conducted for reaffirmation. Seven professional EoL caregivers were chosen to cover the four areas of clinical, nursing, social services, and spiritual care. By covering the spectrum of PC professionals, we affirm the richness and thickness of the interview data as explained in the qualitative research study design literature.^25^ Thickness is affirmed by covering all layers of PC, including at least one professional from each aspect of PC, clinical, nursing, social work, and spiritual care. Each interview, when coded independently, contributed diverse interpretations within the same theme, thus assured the richness of the data quality. This is noted in qualitative research as reaching sample specificity.^26^

Meaningful interpretations (themes) were assigned to codes, using open and axial coding. Coding accuracy was confirmed by comparing data coded by two researchers independently. The consensus was obtained at an open meeting, where key themes that emerged from the coded data were identified. The structure of this article is organized according to the themes that emerged from PCHP data and corresponding plots. This was performed bearing in mind the aim of the article is to uncover major themes that emerged from PCHP encounters in delivering PC to ethnically diverse SA patients. To compare with the FC views from the perspectives of care receivers, the corresponding text (plots) from FCs for the same theme that emerged in PCHP data analysis was discussed. The following interpretation brings out the dual perspectives that were portrayed within the same theme. Following a discussion of the literature, recommendations are formulated on how to reach common grounds to provide integrated EOL health and social care delivery to ethnically diverse SA populations.

### Theoretical frameworks and methodological approaches

The constant comparison method is noted as a critical part of the grounded theory approach to qualitative data analysis.^23^ The application of the method, for this study data, differentiates contradictory/conformity perceptions between the PC team members and FCs. A study that applied the same method differentiated PCHPs’ and FCs’ perspectives in choosing the place of death in Belgium.^8^ In this research, the constant comparison method is used within the themes grounded in PCHP data to illustrate the concordance and discordance of the perspectives between PCHP and FC narratives.

The constant comparison method followed the four-stage approach described by Fram.^27^ First, using a narrative analysis of the interview data, the perceptions and experiences of PCHPs were systematically explored, and emerging themes were identified using the grounded theory approach.^23^ The corresponding codes that came from different PCHPs (with differing roles in PC) under the same theme were organized. In the third step, the parallel codes from the FCs’ responses to the same encounters were collated. In the fourth step, a discordance–concordance analysis was conducted comparing the codes of EoLC deliverers and consumers, namely the PCHPs and FCs. Finally, in the discussion, the interpretation of findings was compared with the existing literature-based evidence.^23^

No studies have focused on comparing ethnically diverse caregivers’ EoL experience with that of Western professional caregivers using discordance–concordance analysis of the primary data collected from both types of caregivers. The concordance–discordance classification has been applied in synthesizing healthcare providers’ and consumers’ perceptions in two EoLC literature reviews. A scoping review that included 21 qualitative studies revealed a mixture of concordance and discordance of perspectives between three parties: patients, professional care providers, and FCs.^5^ Another narrative literature synthesis included 39 studies on EoLC to find factors contributing to concordance and discordance between family and patient EoLC preferences and decision-making. They found relational conflict and lack of awareness as contributing factors to discordance in EoLC preferences.^22^ These methods that have performed well in EoLC research data analysis shaped the theoretical framework of our study.

## Findings

### Demographic characteristics of the participants

The PCHP sample was primarily women (54%). Nurses made up 14%, physicians 28%, social workers 14%, and the balance of the PCHP participants were spiritual care workers and health educators (44%). All had working experience in PC between 1 and 16 years (average: 8 years), and all of them had experience delivering EoLC to SA patients, but not necessarily to the same individuals given care by the FC participants. Owing to ethical considerations, we were unable to make the direct link.

The SA population in Halifax is small, and to preserve anonymity and confidentiality, the demographic characteristics of the FC sample are summarized using percentages. The sample was made up of 28% female caregivers, married (100%), an average of 50 years of age, had children, and the majority were living with children. Their native languages included Bengali, Urdu, and Tamil. All participants had postsecondary education from their country of origin, and their incomes fell above the Canadian median income level (US$61,348).^28^ Most participants were from India (70%), and others were from Sri Lanka and Bangladesh. Their religious affiliations included Hindu (70%) and Catholic (30%). They have given EoLC to a family member (deceased husbands, wives, mothers, and fathers) within the last 4 years on average (range: 1–7 years). All seven patients were deceased from cancer, kidney/liver failures, and heart problems and were mentally capacitated during the time they received EoLC.

### Thematic analysis findings

The four themes that emerged from the PCHP data and relevant codes from FCs displayed in Table 2 have no one-to-one correspondence. But exact quotes and short expressions have been used to give a voice to both groups of caregivers. In what follows, plots were used to illustrate the meaning assigned to codes from PCHPs’ and FCs’ expressions.

**Table 2.** Emergent themes and codes.

Theme	Professional caregiver	Family caregiver
The meaning assigned to palliative care	- Caring for within the last few months of life - Providing/managing good quality of life at the EoL - Providing grieving support to the family - Managing symptoms - Controlling pain - Listening to the patient (respecting autonomy)	- Once he is here, he won’t go back home - Keeping in quarantine (isolation) - Do not know, we do not use it (palliative care). - Waiting to die - Taking a rest - (Health professionals) Giving up on the patient
Role of family in patient-centered EoLC delivery	Understanding and acting according to the following: - No treatment to prevent death - Based on the patient’s interests - Prolonging life treatment is inappropriate - Supporting family for future - Rendering physical care	- Insisting diagnostic testing - Insisting force-feeding - Helping physical care (bathing, lifting, and moving) - Praying to relieve pain - Employing a mediator between family caregiver, patient, and healthcare professional
Communication and disclosure of prognosis	- Indirect communication through a family member - Expecting Canadian etiquette - Language barriers - Privacy concerns with the same ethnic group members - Respectful of autonomy and being truthful to the patient	- Public closure of the truth about the disease condition - Language barriers - Direct communication between patient and the healthcare professional - Conceal critical health information from the patient
Patient-centered EoLC decision-making	- Ensuring equity across diverse cultures - Working through fluidity and adapting to the situation - Being mindful and respectful of the culture and family - Integrating family into decision-making - Considering nuances in each culture	- Leaving things based on God’s wish to happen - Patient is independent in making decisions - Situation dependent - Collective (family) decision-making by a dominant family member

EoLC, end-of-life care.

### The meaning assigned to PC

The PCHP assigned meaning to PC is tied to their professional role. Nurses defined PC as facilitating the EoL process and physicians defined PC as the process of managing symptoms and quality of life. To social workers, PC meant bridging the two types of caregivers, to ensure family needs are met, and to spiritual care workers, it is fulfilling patients’ and families’ desires and religious needs.

Palliative care is caring for people who are dealing with the last few months of their life. – PC nurse

It is the process of helping people who have an advanced illness, for whom the goal is no longer cure but simply the management of symptoms, while adapting their lives to their situation, so that they can keep living the best possible quality of life for as long as they can. – PC physician

We found a divide among FCs in their understanding of PC. One group’s understanding of PC was providing care at the EoL. They thought ‘Once he is here, he won’t go back’ and the patient is ‘Waiting to die’. These were the FCs with higher levels of education and acculturation who had a better understanding of PC. One FC stated,

PC is the healthcare given in the palliative care unit while taking a rest – FC

The meaning assigned by this group is somewhat consistent with the notion held by the PCHP team. In what follows, this group of FCs is noted as EoL-accepted group.

However, the meaning assigned to PC by another group of FCs discorded the perception of PCHPs (Table 3). For one FC (wife), PC meant going to the hospital to take rest and thus anticipated for the patient to come back home alive. Yet another FC (husband) thought his wife was quarantined because they just migrated to Canada. This group of FCs (in what follows noted as EoL denial) entertained the hope that the patient would survive after receiving PC. Within this notion, discontinuation of treatment was viewed negatively as health professionals giving up on the patient.

**Table 3.** Meaning assigned to palliative care.

Type of caregiver	Comments
Palliative care physician	‘The classic definition is that this is the comprehensive, total care of patients facing the end-of-life- or life-threatening illnesses where symptom management and quality of life become the paramount factors and that treatment decisions or not treating are actually filtered through the patients’ values system and their understanding of their disease [spectrum]. It is a process and not an event and needs an ongoing dialogue about their values’.
Pastoral care	‘Palliative care area that I am working in is grief and bereavement. Palliative care is providing good quality of life to the patient at end of life and for families afterward providing support in their grief’.
Family caregiver 1 (EoL denial)	‘It [the palliative care] means once he’s here, he is not going back. I was shocked. I said, “Nobody told me that, I just came here thinking he is taking a rest, and after that, he would go back.” But then he was on intravenous and after all those things happened made me surprised and upset’.
Family caregiver 2 (EoL accepted)	So from that point of view, if you ask, yes we knew that she is not going to last.

EoL, end-of-life.

### Role of family in a patient-centered EoLC delivery

At this imminent EoL stage, PCHPs expected the family to be prepared for the death to happen on any day (Table 4), not to bear unrealistic expectations of medical miracles to happen to prevent death and not to anticipate the patient to live one more day. The obscurity of survival expectations manifested in making undesirable, and to some extent, unreasonable therapeutic demands, made by FCs, who were not competent to advise on EoLC clinical decisions.

**Table 4.** Role of the family in the patient-centered EoLC.

Type of caregiver	Comments
Palliative care physician 1	‘You are telling people [family members] that at one point, we are expecting death to happen. And we don’t have any treatment to bring [patient] him or her back. But, you know, there is an expectation on the part of the society as a whole that we can keep pushing back the values of what is a preventable death’.
Palliative care physician 2	‘. . . people had very different opinions about what we were actually doing, how appropriate it was?, What we were accomplishing?. Who was driving the decision-making? And were the decisions that were being made in his [patient] best interests? And, the concern was that if the interventions that were ongoing were being driven by the family who wasn’t competent to make the type of decisions that were being made and that it was causing him unnecessary discomfort, with the goal of making the patient live just one more day, just one more day deem inappropriate’.
Palliative care nurse	‘Well, they [FCs] tend to interfere too much. I think they were pretty distressed and pretty overwhelmed. One patients wife wanted to do things well by herself and by her children for her husband [patient]. You know, when they came in here, all the physical care was taken care of. You can just be the family member’.
Family caregiver 1 (EoL denial)	‘After the radiation treatment, I just wanted to have another MRI just to see his improvement, but they [healthcare professionals] wouldn’t do it – “no, no, no – there’s no need for that”. And I said, “no – he’s gone through that much, and I want to see the improvement, and that you find out through the MRI”. So by force, they did the MRI’.
Family caregiver 2 (EoL accepted)	‘I always asked one thing to God – please don’t let him suffer [with pains] or anything. . . that is another part that I used to do. [It came to] he never had any pain. I was quietly doing my prayers, and nobody knew what I was doing . . .’

EoL, end-of-life; EoLC, end-of-life care; FCs, family caregivers.

They should not have rigid rules. They should change the rules according to the circumstances. Like when I said ‘His stitches are bleeding, please can anybody vacuum?’, and they said ‘no’. – EoL-denial FC

These demands were discordant with the fundamentals of the PC process followed by PCHPs, in situations when cessation of curative treatments was found futile, and the focus was placed on comfort care as a core function of EoLC (Table 4). The PC nurses noted, FCs insisting on aggressive treatment, designed for disease worsening prevention and cure, (Table 4) as interfering with comfort care. In their view, comfort care deems necessary to maintain quality of life and symptom and pain management. A collaborative FC role expected by PCHPs includes rendering support to fulfill nontherapeutic EoLC needs, by answering PCHPs’ questions to make the patient’s life comfortable and ensuring patients’ wishes are met.

Family caregivers’ role is to answer questions about what support is available to them and participate to making things as good as they can be and that they can do preparations for the future. – PC nurse

However, the FCs, who held unrealistic expectations of ‘the future’, were ineffective in collaborating in their involvement in the EoLC delivery. PCHPs expected FCs to facilitate culturally appropriate food and clothing needs, attend to personal care without crossing professional boundaries, and not interfere with the clinical course of EoLC (Table 4).

Our constant comparison reveals these FCs who crossed boundaries were in the group that denied EoL in their conceptualization of PC. This death-denial group of FCs forced the continuation of aggressive therapy, insisted on expensive diagnostic imaging, and force-feeding manually, even when the patient has lost appetite. These FCs considered avoiding these demands by PCHPs as giving up on the patient and discontinuing aggressive treatment (e.g. chemotherapy) was perceived as letting the patient die.

The second group of FCs, those who accepted EoL, were fulfilling PCHPs’ expectations ‘quietly’ using a low-key collaborative approach (Table 4) and gained pleasure from fulfilling filial duties.

I prepared breakfast for her, myself. We both had it and this was her last day. And I was the one who gave her all the medication including morphine. – FC

Some FCs in this death-accepted group employed mediators, often an extended or immediate family member with healthcare knowledge, to further facilitate the family involvement in EoLC, and this commitment minimized the interference with optimal EoLC delivery.

One FC said, ‘My daughter, she was always on the cell phone talking to[them] . . . and she got feedback from them and on the basis of that feedback, she told me what the best thing is to do for her [the patient]’.

### Communication and disclosure of prognosis

The PCHPs respected patient autonomy, disclosure, and independent decision-making and allowed mentally capacitated patients to receive prognostic information on their request.

As one PCHP noted, ‘We are compelled to tell the truth to the patients if they ask directly what is wrong with me’. – PC physician

This notion of truth revealing went against FCs’ expectations to conceal critical personal health information from the patient. PCHPs acted according to FCs’ wishes of closure, only when patients refused information receipt and otherwise requested to filter through family members.

As per the comment by one PCHP ‘. . . probably [I’m] being unethical by forcing information down someone’s throat [Meaning the patient] who has chosen to have that filtered through another member of the family’. – PC physician

Bereavement counselors espoused the same view: ‘The older South Asian people do not want any communication about their illness directly with them, but to talk with their family. And that was definitely a cultural thing’.

Within this premise, the EoL-denial FCs were found discourteous beyond accepted Canadian etiquette, and PCHPs noted this behavior as a way of expressing caregiver frustration (Table 5). Other PCHPs attributed this communication style to caregiver stress that drove them to use an authoritative tone to demand futile therapies, and to lack of English proficiency skills to understand PCHPs’ interpretation of the prognosis of imminent death. The EoL-denial group of FCs considered disclosure, of a definitive timeline of death, to the patient, as deemed inappropriate (Table 5). Instead, information filtering through a dominant family member, in the absence of visitors, before disclosing directly to the patient, was preferred. This way the ‘bad news’ can be cushioned, and invading privacy can be avoided (Table 5). FCs, who were conversant in English, found no communication issues and perceived the lack of acculturation to Canadian etiquette by immigrants, which may have resulted in undesirable communication issues.

**Table 5.** Communication-related comments.

Type of caregiver	Comments
Healthcare professional (nurse)	‘But if there is a person swearing at us or dealing with us in a way that we are not used to . . . I think she felt she was in charge and she had to be directive with the people who were coming to her home. She wouldn’t ask for things. She would tell me that I had to do it. She didn’t say “please” or “thank you” or “would you mind” or anything like that. I mean, it was her. It is not the time to be coaching people on etiquette, Canadian etiquette or etiquette with health care workers or you know my right to be treated with respect’.
Family caregiver (EoL denial)	‘Should have asked his wife to stay, and the other person to leave. This is a personal thing, you know – I didn’t like it. I’d rather feel that way, like gradually I like to tell, just like in one shot . . . I didn’t want that he should go through with this . . . maybe slowly, politely, in a different manner . . .’
Family caregiver (EoL denial)	‘And I said “you came to my house, and you told me he’s going to live only 2 weeks. That made me very upset. By the way, you are not a God. Number two, I don’t want you to tell that kind of statement to other patients.”’
Family caregiver (EoL accepted)	‘The only thing is that people who do not speak English well or are new to this country, who don’t have that kind of exposure that we had, they don’t get good care. They don’t know how to ask questions or get advice. And the doctors or other professionals don’t know what kind of questions to ask and how much to explain’.

EoL, end-of-life care.

### Patient-centered EoLC decision-making

PCHPs followed an open-minded patient-centered approach as one physician explained:

There are certain things to be addressed, decisions to be made, for everybody. But you can’t go in with a prescribed decision. – PC physician

They personalize the process of EoLC decision-making, with a focus on patient-centered care (Table 6). The strategy used by the PCHPs was to quickly assess FC’s beliefs, values, and practices to adapt the care plan to meet their expectations.

**Table 6.** Patient-centered EoLC decision-making.

Caregiver type	Comments
Pastoral caregiver	‘With some people you have to be very quiet, some people ask a lot of questions, some people like humour, some people want to be very formal, some people want to be very casual, some people want you to be very direct and upfront with their illness, some people want to sort of, tread around it and they know it is there but they don’t want to be in their face’.
Palliative care physician	‘And how can I change what I would normally do is to meet their needs. So, making a deliberate effort to acquire that knowledge and integrate that into the care plan and ensure that other people are aware of that. I would never pretend or presume that I knew everything there is to know about a particular religion or culture. I try to avoid stereotyping. How do I need to adjust the way I provide care to meet their needs? I do that with everybody anyway because everybody’s situation is different. I don’t want to offend anyone. But I don’t want to overlook something either’.
Family caregiver (EoL denial)	‘Basically, we made the decision with my daughter. She was very close to my wife’. ‘. . . it becomes like a melting pot . . . there are unspoken truths and unspoken understandings. We made decisions collectively even toward the end’.
Family caregiver (EoL accepted)	‘She was well educated about her disease. My wife [the patient] would make the decision on her behalf after consulting doctors, outside our culture or religion. She was very well informed. She knew a lot of things. She always wanted to be listened to. She was very much into it’.

EoL, end-of-life; EoLC, end-of-life care.

So, I need to be able to get a reasonably quick read of how I am going to approach this decision about care with this family. – PC physician.

This adaptation provides leeway to institute collaborative patient- and family-centered decision-making, especially in setting up the EoL care plan (Table 6). The bereavement counselors stated the way of adapting to the ethnic and gendered variations is to move beyond the dominant cultural values as the single reference point. The way to gauge cultural variants that drastically vary from the single reference point was not to use rigid tactics and maintain fluidity. These are covalues of patient-centered care.

I think we have to get past the reference point, stepping back and have a basic approach that acknowledges and respects the culture, then that leaves you open to learning about that individual’s culture. – PC nurse.

This approach was seen as the best approach to making culturally appropriate patient-centered EoLC delivery decisions.

Cultural variations exist even within a single ethnic group. The EoL-denial group of FCs believed in a divine determined path to destiny, and they perceived PCHPs deciding to discontinue life-sustaining treatment as going against the will of God. The clinical decisions on proceeding with comfort care, directed in the presence of life-limiting conditions were viewed by FCs as distorting clinical course. This group of FCs’ participation in the EoL clinical decision-making process was obstructed by the expectation of making every attempt to make the patient live one more day, by insisting on futile therapeutics, expensive diagnostic imaging, and force-feeding orally.

Nevertheless, those EoL-accepted FCs and/or patients acculturated to the Western biomedical culture prefer independent decision-making. In those circumstances, FCs’ decision-making was circumstance driven, irrespective of making ties to their ethnic origin.

My wife made her [patient] own decisions outside our culture or religion. Your immediate environment has more impact on you than the rest of xxxx [country of origin] has. – FC

**Figure 1.**
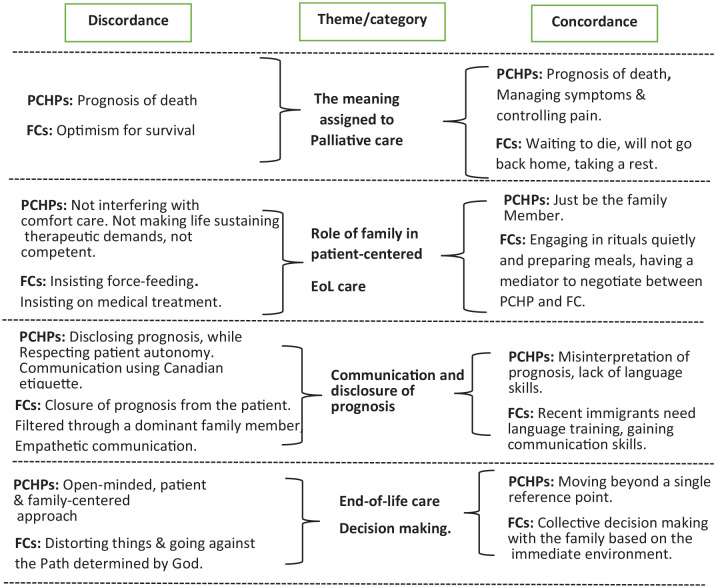
Summary of the main themes featuring discordance and concordance. FCs, family caregivers; PCHPs, palliative care health professionals.

This immediate environment was facilitated by extended family involvement in collaborative decision-making, which these FCs identified as collective decision-making with close relatives. The results of the key features of the discordance–concordance analysis are summarized in Figure 1.

## Discussion

This is the first study that explored discordance and concordance of EoLC-related experiences, perceptions, and encounters of SA FCs and PCHPs, which occurred in the same inpatient care setting. While uncovering the variants of perceptions among FCs, and between FCs and PCHPs, the study findings weaved the discordances and concordances of meaning assigned to PC to three areas of PC: the role of the FC, communication needs and challenges between FCs and PCHPs, and barriers to FC participation in EoLC decision-making. Our study uncovered two groups of FCs, demarcated by acceptance or denial of EoL, and participating differently in their EoLC giving. The study findings pinpointed directions toward the integration of FCs, with similar and opposing perceptions to that of healthcare professionals, into PC planning-related decision-making. In the following discussion, our study findings on SA FCs in Canada and Canadian PCHPs’ perceptions and encounters, in inpatient care settings, are compared with the existing EoLC research-based evidence, coming from other settings, countries, and ethnicities.

Among the four themes that emerged in PC health professionals’ discussions, EoL-accepted group of SA FCs concorded with PCHPs, understood that FC’s role of involvement should be confined to filial duties, recognized communication deficiencies, and understood the need for FCs to corporate in collective decision-making based on the immediate environment (Figure 1).Within the context of the same four themes, EoL-denial FC group was optimistic about prolonging survival, refuted comfort care, denied disclosure of prognosis, and EoL decision on switching to comfort care was framed as going against the divine determined path. A scoping review examined the discordance–concordance of experiences between patients and their caregivers (professional and family) using research findings from 11 countries, which included 1 from Canada.^5^ They included articles published on patients older than 60 years and dying within the same year. They found concordance between patients and healthcare professionals on the need for patient-centered communication, especially around prognostic uncertainty.^5^ Our findings from EoL-accepted SA FCs eluded similar communication issues, and thus, communication of prognostic uncertainty may reflect the same notion across different ethnicities and care settings. Although the multicountry review study included only one Asian study, from Hong Kong, it indicated Chinese patients’ reluctance to receive EoL prognostication as a ‘bad omen’.^5^ This may explain our study’s SA FCs’ request to filter through information from them before disclosing it to the patient.

The divergent understanding of EoL was prominent among SA FCs, who were optimistic about patient survival, denied the prognosis of EoL, bore conflicting views and expectations, and perceived competing interests in EoLC delivery practices compared with these of the PCHPs. Their caregiving role, communication with PCHPs, and participation in the decision-making process were different from those FCs who accepted the prognosis of imminent death. Eventually, these EoL-accepted FCs met PCHPs’ expectations of carrying out filial duties. A US study on a mixed ethnic sample of caregivers described the denial of the prognosis of death (occurred among 28% discorded with PCHPs), by FCs, and they described this as a religious-based way of maintaining optimism for recovery to give blessings to the patient. They further mentioned this is one way for the FC to get emotional comfort.^29^ The EoL-denial SA FCs, in our study, entertained the hope for survival and this optimistic bias was noted in the Asian EoL literature as a common practice in this ethnic group.^30^ We further revealed a consequence of this optimistic bias, as creating dissatisfaction toward the EoLC delivery system when the PCHPs prognosticate imminent EoL.

The EoL-denial group of SA FCs, in our study, entertained survival optimism that resulted in interfering comfort care delivery, demanded the closure of prognosis from the patient, and was unable to fully participate in clinical decision-making with PCHPs. A scoping review of 15 qualitative studies that included SA and other ethnic groups reported the reason for the denial of imminent death by FCs was because upholding the view of believing and speaking of death would evoke death.^1^ They indicated the need for PCHPs to attend to their usual aspects of care while acknowledging the hope, encouragement, and optimism held by the FCs.^1^ Paying particular attention to SA culture and inpatient EoLC, our findings suggest the need for PCHPs to move beyond dominant cultural values and practices. This was confirmed by another study among PCHPs, who worked in community PC settings in the United Kingdom that focused on culturally competent PC for SAs. They suggested incorporating the cultural needs of the SA collective unit, patient, family, kin, and community.^31^ Our findings on the need to incorporate cultural needs from inpatient care settings in Canada echoed what is found in the community setting in another country.

The EoL-denial SA FCs acted as gatekeepers, crossed boundaries beyond professionals’ expectations, and misconstrued patients’ right to know the truth. While in denial of the truth of imminent death, they expected to conceal the truth from the patient. A review paper of six multiethnic EoL research studies concluded the integration of family early into care planning is a solution to minimize FCs’ interference in institutional care regimes.^32^ Although our study findings affirm the necessity for ethnically diverse family involvement, their stress-induced negative interactions impede participation in the optimal EoLC delivery process. A US study revealed the reason for becoming emotionally charged by Chinese FCs is experiencing burnout by carrying out filial duties; nevertheless, another study noted that Chinese caregivers consider filial duty as a motivator.^33,34^ Even the Chinese FCs living in Hong Kong experienced similar complex interactions and contested priorities with the Chinese PCHPs.^35^ These findings may suggest Asian FCs, carrying out filial obligations at EoL, as having contesting priorities with PCHPs, despite their ethnic origin, regardless of the place of living, home, or abroad. Another US study indicated families are too fatigued to make important EoL decisions.^36^ Although the FCs’ involvement in EoLC is found challenging across ethnicities and geographies, the benefits of their involvement are evident. A multiethnic research review concluded that family integration into EoLC planning should be considered as means to minimize individual and shared challenges that arise across ethnicities and geographies.^32^

The EoL-denial FCs in this study refuted direct communication of (1) the prognosis of imminent death with the patient and (2) timelines of death to happen with patients and FCs. We found four reasons for the communication challenges of prognosis: disparities between Western biomedical ethics and practices of disclosure (as means of respecting autonomy), patient and family cultural values, not being fluent in English, and not being emersed in Canadian etiquette. Family values are culturally embedded, and the solution for the direct communication challenges was to use a family member as a mediator.^18^ This cultural practice of preferred indirect communication through a dominant family member that we found was noted in the Asian EoL literature as a result of relational autonomy, which subdues patient and physician authority.^37^ The relational autonomy noted in our study is common to other Asians living in Asia and non-Europeans living in Canada.^37^ Another Canadian study confirmed this relational autonomy when coupled with the language barrier impedes effective pain and symptom management in non-European patients in EoLC.^38^ So relational autonomy is not unique to SAs living in Canada. Literature suggests the involvement of the primary care physician, someone who has established a trusted relationship with the patient and the family, as a well-suited person to be involved in EoLC planning and underscored the practice of empathetic communication style by all parties.^39^ The primary care physician, also known as the family doctor in Canada, would not have the same relational autonomy as the dominant family member. Another solution suggested by English-fluent FCs in our study emphasized the need to use the right language of inquiry to get satisfying solutions. It is paramount for immigrant service providers to expand their language services to include health communication skills at the time of arrival to Canada.

This study revealed a two-prong approach to patient-centered decision-making used by PCHPs; first, learning from the patient and family on their needs and cultural preferences and second, responding to EoLC issues without using dominant Western biomedical culture as the reference point. The first approach covers the cultural norms explained in healthcare as ‘learned, shared and transmitted values, beliefs and norms’.^40^ Supportive care professionals, namely social and spiritual care workers, in our study, indicated patients and providers must navigate the differing expectations, perspectives, and preferences in EoLC decision-making, without a single reference point. A US study suggested cultural variation in EoLC should be broadly assessed within the context of the patient’s unique history, family structure, and social status.^41^ Therein, FCs and PCHPs must maintain cultural fluidity in EoL decision-making. This notion of cultural fluidity was maintained by FCs who accepted imminent death and made decisions based on the immediate environment. But EoL-denial FCs maintained cultural rigidity instead and involved their family members with health education as mediators, to facilitate the EoL decision-making process. In health behavior theories, culture is viewed as a complex dynamic process, influenced by social, historical, geographic, and political forces.^42^ This dynamic process was redefined by FCs who accepted EoL, in our study, by admitting that the immediate environment has more impact on EOL decision-making than their inherited culture. They accepted the prognosis of EoL and collaborated with clinical decision-making on comfort care and pain management. EoL-denial FCs insisted on invasive treatments and care and inhibited participation in clinical decision-making on patient-centered comfort care planning. Making things more complicated is the divergent understanding of comfort care, such as the administration of sedatives as incapacitation of patients’ sense of control which limits much-needed spiritual and social engagement.^5^ Researchers suggested reinforcing intensive education, on the futility of life-sustaining treatments at the EoL stage and the purpose of comfort care, using diverse cultural contextualization before EoLC decision-making.^5^

Based on the constant comparison of findings, from the literature and our research findings, in the discussion, the following recommendations are made with a focus on the integration of FCs into EoLC planning and decision-making.

**Table:** 

Box 1. Recommendations for health systems.
• Gaining conceptual clarity of PC by ○ Ongoing dialogue, ○ Optimizing informal in-person communication with FCs, ○ Use South Asian FCs who understand PC as ambassadors. • Optimizing family caregiver engagement in EoLC delivery: ○ PCHP to acknowledge and be educated on filial duties and obligations of FCs from different cultural communities, ○ Integration of family early into the care plan after gaining conceptual clarity, ○ Educate FCs on prognosis, step-by-step process of dying in lay terms, ○ Educate FCs on expected role play in patient-centered care in each of these steps, ○ Educate FCs on harm incurred to the patient in force-feeding and administering invasive therapies. • Establishing communication channels and standards: ○ Immigrant language service providers to include Canadian etiquette and healthcare ethics and practices in their lesson plans delivered at the time of arrival, ○ Involve primary care physicians and family members with healthcare knowledge as mediators to facilitate communication, ○ Educate FCs on EoLC ethics and practices on disclosure and truth at the beginning of EoL planning using appropriate channels. • Patient- and family-centered decision-making: ○ Involve family in decision-making of discontinuation of invasive therapies, manual feeding or tube feeding, and resuscitation orders, ○ Maintain cultural fluidity without a reference point, ○ Culturally contextualize comfort care and pain management plan, ○ Educate FCs on the EoLC plan.

EoL, end-of-life; EoLC, end-of-life care; FCs, family caregivers; PC, palliative care; PCHP, palliative care health professional.

### Limitations

First, our study sample was small and included seven SA FCs and seven PCHPs. We were unable to find more PCHPs who met the inclusion criteria, especially those who have given inpatient care to an SA patient within the last 10 years, in the small tertiary inpatient PC unit. We succeeded in recruiting at least one PCHP from each of the components of PC, covering clinical, social, and spiritual care. Second, the 10-year span may be too long to recollect encounters for some. But we found it difficult to recruit SA FCs who had faced the recent death of family members and who were willing to share their experiences. Nevertheless, our study findings that came from a single geography and one ethnicity shared some common and other unique perspectives of EoLC needs and expectations from other ethnicities and geographies.

## Conclusion

The PCHPs’ perceptions and encounters of inpatient EoLC delivery to SA patients bore concordant and discordant views and understandings of the FCs. The divergence was rooted in FCs’ denial and convergence was imbedded in acceptance of the EoL. Gaining conceptual clarity on the meaning of PC and the process of EoLC delivery are crucial to successfully integrating ethnically diverse FCs into EoLC planning and decision-making. The establishment of a two-prong learning process, that entails PCHPs learning culturally embedded filial duties and obligations and FCs learning (1) communication standards practiced in healthcare settings and (2) EoLC process and ethics, is recommended. This mutual understanding will (1) reduce divergent views and challenges, (2) optimize FC integration into EoLC delivery, and (3) gain caregiver satisfaction. More research is needed to understand factors contributing to the integration of ethnically diverse patients and FCs into patient-centered EoLC planning and delivery in inpatient care settings.

## Declarations

**Ethics approval and consent to participate:** This research project received ethics approval from the Capital District Health Authority (REB File # CDHA-RS/2007-162), Research Ethics Board, and Dalhousie University Health Sciences Ethics Review Board.

**Consent for publication:** Study participants provided informed consent at the beginning of each interview. Copies of transcribed data were sent to all study participants to facilitate further feedback. One family caregiver indicated a section to be removed and we respected the request.

**Availability of data and materials:** Original data are not available to the public because of ethical reasons. Interview guides and consent forms are available on request.

## References

[1] Bosma H Apland L Kazanjian A . Cultural conceptualizations of hospice palliative care: more similarities than differences. Palliat Med 2010; 24: 510–522. 19910394 10.1177/0269216309351380

[2] Meghani SH Levoy K Magan KC , et al. ‘I’m dealing with that’: illness concerns of African American and White cancer patients while undergoing active cancer treatments. Am J Hosp Palliat Care 2021; 38: 830–841. 33107324 10.1177/1049909120969121 PMC8424597

[3] Steinhauser KE Christakis NA Clipp EC , et al. Factors considered important at the end of life by patients, family, physicians, and other care providers. JAMA 2000; 284: 2476–2482. 11074777 10.1001/jama.284.19.2476

[4] Swami M Case AA . Effective palliative care: what is involved? Oncology 2018; 32: 180–184. 29684230

[5] Carlini J Bahudin D Michaleff ZA , et al. Discordance and concordance on perception of quality care at end of life between older patients, caregivers and clinicians: a scoping review. Eur Geriatr Med 2022; 13: 87–99. 34386928 10.1007/s41999-021-00549-6 PMC8359918

[6] Bullock K . The influence of culture on end-of-life decision making. J Soc Work End Life Palliat Care 2011; 7: 83–98. 21391079 10.1080/15524256.2011.548048

[7] Isıkhan V . The place and future of social work in palliative care services in Turkey: state of the art. Soc Work Public Health 2017; 32(3): 192–201. 27976990 10.1080/19371918.2016.1230080

[8] Reyniers T Houttekier D Cohen J , et al. The acute hospital setting as a place of death and final care: a qualitative study on perspectives of family physicians, nurses and family careers. Health Place 2014; 27: 77–83. 24577161 10.1016/j.healthplace.2014.02.002

[9] Temel JS Petrillo LA Greer JA . Patient-centered palliative care for patients with advanced lung cancer. J Clin Oncol 2022; 40: 626–634. 34985932 10.1200/JCO.21.01710

[10] Arora NK Gayer C DiGioia K , et al. A patient-centered approach to research on palliative care for patients with advanced illnesses and their caregivers. J Pain Symptom Manage 2017; 54: e1–e9. 10.1016/j.jpainsymman.2017.06.012 28803084

[11] Statistics Canada. Immigration and ethnocultural diversity: key results from the 2016 census, 2017, https://www150.statcan.gc.ca/n1/daily-quotidien/171025/dq171025b-eng.pdf

[12] Tatum PE Mills SS . Hospice and palliative care: an overview. Med Clin North Am 2020; 104: 359–373. 32312403 10.1016/j.mcna.2020.01.001

[13] Rine CM . Is social work prepared for diversity in hospice and palliative care? Health Soc Work 2018; 43: 41–49. 29244119 10.1093/hsw/hlx048

[14] Sepúlveda C Marlin A Yoshida T , et al. Palliative care: the World Health Organization’s global perspective. J Pain Symptom Manage 2002; 24: 91–96. 12231124 10.1016/s0885-3924(02)00440-2

[15] Milberg A Olsson EC Jakobsson M , et al. Family members’ perceived needs for bereavement follow-up. J Pain Symptom Manage 2008; 35: 58–69. 17949942 10.1016/j.jpainsymman.2007.02.039

[16] Tadic ELA Sw M Achelle R , et al. Rôle des travailleurs sociaux dans les équipes interprofessionnelles de soins primaires [The role of social workers in interprofessional primary healthcare teams]. Healthc Policy 16(1): 27–42. 32813638 10.12927/hcpol.2020.26292 PMC7435073

[17] Chan RJ Webster J Bowers A . End-of-life care pathways for improving outcomes in caring for the dying. Cochrane Database Syst Rev 2016; 2: CD008006. 10.1002/14651858.CD008006.pub4 PMC6483701 26866512

[18] Weerasinghe S Maddalena V . Negotiation, mediation and communication between cultures: end-of-life care for South Asian immigrants in Canada from the perspective of family caregivers. Soc Work Public Health 2016; 31: 665–677. 27362293 10.1080/19371918.2015.1137521

[19] Fang ML Sixsmith J Sinclair S , et al. A knowledge synthesis of culturally- and spiritually-sensitive end-of-life care: findings from a scoping review. BMC Geriatr 2016; 16: 107. 27193395 10.1186/s12877-016-0282-6 PMC4872365

[20] National Association of Social Workers. NASW standards for palliative & end of life care, 2004, p. 31, https://naswpress.org/product/53608/nasw-standards-for-palliative-amp-end-of-life-care

[21] Hui D Bruera E . Models of palliative care delivery for patients with cancer. J Clin Oncol 2020; 38: 852–865. 32023157 10.1200/JCO.18.02123 PMC7082156

[22] Mulcahy Symmons S Ryan K Aoun SM , et al. Decision-making in palliative care: patient and family caregiver concordance and discordance-systematic review and narrative synthesis. BMJ Support Palliat Care. Epub ahead of print 22 March 2022. DOI: 10.1136/bmjspcare-2022-003525. PMC10804031 35318213

[23] Glaser Barney G Strauss AL . Developing grounded theory: strategies for qualitative research. Chicago, IL: Aldine Publishing, 1967.

[24] Weerasinghe S Mitchell T . Connection between the meaning of health and interaction with health professionals: caring for immigrant women. Health Care Women Int 2007; 28: 309–328. 17454180 10.1080/07399330601179794

[25] Fusch PI Ness LR . Are we there yet? Data saturation in qualitative research. Qual Rep 2015; 20: 1408–1416.

[26] Malterud K Siersma VD Guassora AD . Sample size in qualitative interview studies: guided by information power. Qual Health Res 2016; 26: 1753–1760. 26613970 10.1177/1049732315617444

[27] Fram SM . The constant comparative analysis method outside of grounded theory. Qual Rep 2013; 18: 1–25.

[28] Statistics Canada. Income highlight tables, 2016 census. Median household total income and after-tax income by household type, 2017, https://www12.statcan.gc.ca/census-recensement/2016/dp-pd/hlt-fst/inc-rev/Table.cfm?T=108&CMA=001&S=86&O=A

[29] White DB Ernecoff N Buddadhumaruk P , et al. Prevalence of and factors related to discordance about prognosis between physicians and surrogate decision makers of critically ill patients. JAMA 2016; 315: 2086–2094. 27187301 10.1001/jama.2016.5351

[30] Zier LS Sottile PD Hong SY , et al. Surrogate decision makers’ interpretation of prognostic information: a mixed-methods study. Ann Intern Med 2012; 156: 360–366. 22393131 10.1059/0003-4819-156-5-201203060-00008 PMC3530840

[31] Owens A Randhawa G . ‘It’s different from my culture; they’re very different’: providing community-based, ‘culturally competent’ palliative care for South Asian people in the UK. Health Soc Care Community 2004; 12: 414–421. 15373820 10.1111/j.1365-2524.2004.00511.x

[32] Schulman-Green D Feder S . Integrating family caregivers into palliative oncology care using the self- and family management approach. Semin Oncol Nurs 2018; 34: 252–263. 30143346 10.1016/j.soncn.2018.06.006

[33] Hunt GG Longacre ML Kent EE . Cancer caregiving in the US – National Alliance for Caregiving. SLIDELEGEND.COM, 2016, https://slidelegend.com/cancer-caregiving-in-the-us-national-alliance-for-caregiving_59ddd9bb1723ddf46c3f4a5c.html

[34] Thai JN Barnhart CE Cagle J , et al. ‘It just consumes your life’: quality of life for informal caregivers of diverse older adults with late-life disability. Am J Hosp Palliat Care 2016; 33: 644–650. 25948041 10.1177/1049909115583044 PMC4636480

[35] Ho AHY Luk JKH Chan FHW , et al. Dignified palliative long-term care: an interpretive systemic framework of end-of-life integrated care pathway for terminally Ill Chinese older adults. Am J Hosp Palliat Care 2016; 33: 439–447. 25588584 10.1177/1049909114565789

[36] Candrian C Tate C Broadfoot K , et al. Designing effective interactions for concordance around end-of-life care decisions: lessons from hospice admission nurses. Behav Sci 2017; 7: 22. 28420191 10.3390/bs7020022 PMC5485452

[37] Cheng SY Lin CP Chan HYL , et al. Advance care planning in Asian culture. Jpn J Clin Oncol 2020; 50: 976–989. 32761078 10.1093/jjco/hyaa131

[38] Chan A Woodruff RK . Comparison of palliative care needs of English- and non-English-speaking patients. J Palliat Care 1999; 15: 26–30. 10333661

[39] Roth AR Canedo AR . Introduction to hospice and palliative care. Prim Care 2019; 46: 287–302. 31375182 10.1016/j.pop.2019.04.001

[40] Leininger M McFarland MR . Transcultural nursing: concepts, theories, research & practice. 3rd ed. McGraw Hill, 2002, https://www.amazon.ca/Transcultural-Nursing-Concepts-Theories-Research/dp/0071353976

[41] Koenig BA Gates-Williams J . Understanding cultural difference in caring for dying patients. West J Med 1995; 163: 244–249. 7571587 PMC1303047

[42] Kagawa Singer M . Applying the concept of culture to reduce health disparities through health behavior research. Prev Med 2012; 55: 356–361. 22391576 10.1016/j.ypmed.2012.02.011

